# Mind the Gap: Putting Evidence into Practice in the Era of Learning Health Systems

**DOI:** 10.1007/s11606-018-4633-1

**Published:** 2018-08-28

**Authors:** Jeanne-Marie Guise, Lucy A. Savitz, Charles P. Friedman

**Affiliations:** 1 Scientific Resource Center for the AHRQ Effective Health Care Program, Portland VA Research Foundation, Portland, OR USA; 2 Department of Obstetrics & Gynecology, Oregon Health & Science University, Portland, OR USA; 3 Kaiser Permanente Northwest Center for Health Research, Portland, OR USA; 4 Department of Learning Health Sciences, University of Michigan Medical School, Ann Arbor, MI USA

**Keywords:** health care delivery, evidence-based medicine, implementation research, systematic reviews, health information technology

## Abstract

Due to the increasing amount of available published evidence and the continual need to apply and update evidence in practice, we propose a shift in the way evidence generated by learning health systems can be integrated into more traditional evidence reviews. This paper discusses two main mechanisms to close the evidence-to-practice gap: (1) integrating Learning Health System (LHS) results with existing systematic review evidence and (2) providing this combined evidence in a standardized, computable data format. We believe these efforts will better inform practice, thereby improving individual and population health.

## BACKGROUND

Patients expect that the care provided to them will be based on the best available evidence. However, it is widely recognized that, absent directed efforts to bring evidence to practice, many years will elapse before new, validated evidence will actually be integrated into practice.^1^ A kind of magical thinking seems to pervade our society: a belief that creating more and more evidence will in and of itself speed the application of evidence to practice. Investments in “Big Data” in the absence of concomitant investments in appraising, synthesizing, and translating that evidence into practice will not by themselves realize the greatly anticipated effects on individual and population health. Closing the evidence-to-practice gap will require two key elements: (1) the integration of locally and rapidly generated evidence—what we are calling “Learning Health System evidence”—with cumulative and comprehensive systematic review evidence from the peer-reviewed literature and (2) making this combined evidence available in standardized computable forms so it can be efficiently and effectively assimilated to inform practice.

The Learning Health System (LHS), first envisioned by the Institute of Medicine in *Crossing the Quality Chasm* (2001) and re-expressed in 2007,^2^ described the generation of evidence as a by-product of care delivery and application of that evidence to support continuous improvement, evidence-based care delivery, and population management. As such, the LHS concept requires that evidence generation not be an end in itself; efforts to generate evidence must be accompanied by equally emphasized efforts to apply it to improve health. Currently, there are no pathways for harvesting new evidence, produced by LHSs or any other methods, besides publication in peer-reviewed journals. In this report, we argue for the need to integrate all sources of high-quality evidence addressing a particular question—published or not. We also define a LHS broadly as any entity that routinely and continuously seeks to generate and learn from data, for purposes of improving individual and population health. LHS’s can and increasingly do exist in multiple forms and at multiple levels of scale: single-delivery systems such as the Mayo Clinic,^3^ collaboratives such as the High Value Healthcare Collaborative (see http://www.highvaluehealthcare.org/), and national-scale practice-based networks such as PCORnet (the National Patient-Centered Clinical Research Network).^4^

### A Framework for Local and External Evidence Integration

**Figure 1:**
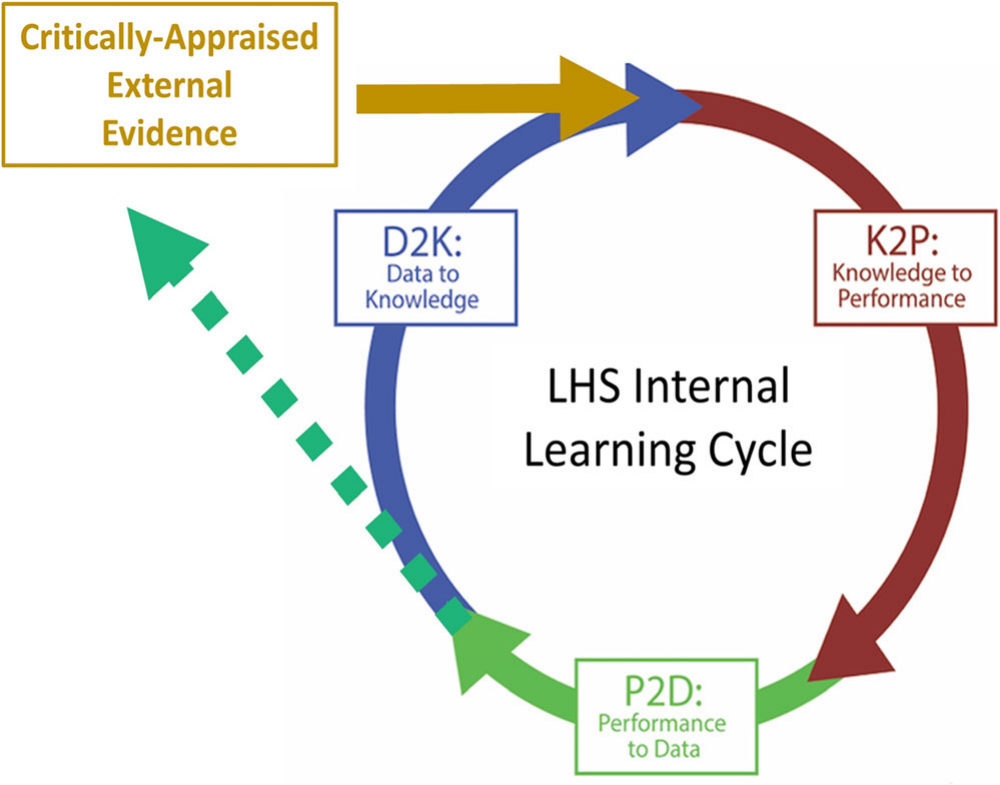
Expanding the cycle of learning through integration of internal and external data and evidence.

While locally generated LHS evidence is clearly important, it is not sufficient. To safely guide practice, local results must combine with the larger body of what is already known about a health problem. What is needed is an expanded cycle of learning that allows “external” evidence from trials, studies, and reviews to inform practice within Learning Health Systems, and conversely, allows data from practice to feed back into the overarching evidence base. In Figure 1, we provide a schematic of the necessary integration of critically appraised evidence into the pool that is needed to guide optimal patient care, as well as the flow of health system data into critical appraisal processes. The power of continuous evidence integration has been demonstrated time and again over several decades. For example, if newly generated evidence were consistently contributed to a cumulative systematic review analysis, thrombolytic therapy for myocardial infarction would have become standard of care 20 years before it became widely used in practice.^5^

Alongside increasing ability for LHSs to generate and analyze their own data—and share the results—producers of systematic reviews are working to tailor both the review process and presentation of results to facilitate bringing evidence to practice.^6,7^ The Agency for Healthcare Research and Quality’s Evidence-based Practice Centers (EPC) program has a long history of engaging patients and other stakeholders and has been engaging health systems through projects to better understand the formats and critical elements that health systems need for evidence that can change practice. The EPC program and the Cochrane Collaboration are also experimenting with methods to improve efficiency of the production of evidence reviews without compromising scientific rigor so that evidence can be continuously updated and available. This sets the stage for integration of external knowledge into LHS activities, as illustrated by the gold arrow in Figure 1, and makes a strong case for representing evidence in computable forms, as will be discussed below.

As illustrated in the dotted green line in Figure 1, organizations that evolve LHS capabilities should be outward looking in putting their data and analytic capabilities to work to contribute to this broader evidence base, even as they become more agile in generating and applying locally generated results. An inward-looking approach where a single LHS generates and uses its own evidence without blending this with what is already known will underperform because it is not making use of everything that is known about a health problem. Moreover, an inward-looking system cannot realize the greater good of making both data and evidence shareable, cumulative, and readily revisable. For many organizations, this will require a shift in culture from viewing data as a closely held asset to one recognizing the value of shared learning by rapidly assimilating data from single-system studies.

### An Ecosystem That Makes Evidence Shareable

New infrastructure will be necessary to support this shift toward near real-time evidence sharing and the routine application of evidence to change practice and improve care through the evolution of LHSs. The initial step in this process is a recognition of the importance of putting knowledge into standardized, computable representations, augmenting varied representations in text, tables, and figures. The importance of this change forms a pillar of the National Library of Medicine’s 2018 Long Range Plan.^8^

Biomedical evidence has traditionally been represented in human readable form—in text, tables, and figures—and published in books and journals. However, knowledge in this format cannot readily promote the mass action required to support the rapid and routine translation of new knowledge to practice, especially given the accelerated pace of new evidence generation in the expanding LHS environment, thus, the urgency for addressing this issue now. Driving improvement with knowledge in human readable form will be limited by access, time, and resources needed to fully inform priority areas for improvement. This current state approach cannot possibly succeed. There is too much entirely new evidence and existing evidence is changing too rapidly.

To enable mass action as we enter the era of LHS, evidence of all types must be available in standardized, computable formats to support the curation, representation, dissemination, and application of evidence. Evidence of all types (rules, recommendations, guidelines, predictive models) can be packaged as digital knowledge objects (DKOs) using a standardized format. Metadata attached to each DKO will describe the knowledge held in the object and will provide essential information about the analytic results used to generate the knowledge, including indicators of the strength of the evidence. These DKOs can be curated and managed in digital libraries and, through mechanisms completely analogous to the traditional function of libraries, made available to a range of users. Taking inspiration from pioneering work in enterprise knowledge management originally performed within Partners Healthcare,^9^ several software products (for example, apervita.com and semedy.com) and standards^10^ that support creation of DKOs and their management in digital libraries already exist. These resources, which will continue to mature, combine to make this vision an achievable reality.

Most important, the modularization of knowledge into DKOs makes possible the integration of locally produced LHS research results and externally published evidence, as envisioned in the previous section. In most straightforward scenario, a health system can generate advice from LHS evidence and published evidence side-by-side, and determine if the advice generated from the two sources conflict or coincide. In more sophisticated scenarios, health systems can create compound DKOs each of which synthesizes the evidence from multiple models. The ability to amass evidence more broadly is important in having sufficient power to support evidence needed to ensure safe and effective care delivery.

To complement the efforts self-organizing, private collaboratives like HVHC, incentivizing a computable knowledge ecosystem will likely require government action such as participation requirements for federally funded projects, organizational priorities for large-scale learning, and training to facilitate clinical use of the most up-to-date indications gleaned for evidence-based medicine. As this ecosystem evolves, the most immediate implication for authors contributing to journals like JGIM will be the extension of the publication pipeline. For papers that result in statistical models, rules, recommendations, or guidelines, publication in human readable form will be followed by representation of this same evidence in a computable form. The details of this approach will evolve over time. As it does, research authors and clinician users of that published research will have important roles to play in designing the pipeline that makes computable knowledge accessible at scale.

## CONCLUSION

We stand on the brink of a transformation in health evidence generation and application, catalyzed by the growth of LHS practices, technological advances, shifts in culture, revised work processes, and a burning “need to know” now. Achieving the greatest benefit from this change begins with recognition that new evidence is necessary but not sufficient for better health and that moving evidence into practice, at present and in the future, will require an ecosystem supporting mass action at an accelerated pace. We must continually mind the knowledge-to-practice gap and take positive steps to bridge it.
